# Evaluating the use of a continuous approximation for model-based quantification of pulsed chemical exchange saturation transfer (CEST)

**DOI:** 10.1016/j.jmr.2012.07.003

**Published:** 2012-09

**Authors:** Y.K. Tee, A.A. Khrapitchev, N.R. Sibson, S.J. Payne, M.A. Chappell

**Affiliations:** a Institute of Biomedical Engineering, Department of Engineering Science, University of Oxford, UK; b Centre for Doctoral Training in Healthcare Innovation, University of Oxford, UK; c CR-UK/MRC Gray Institute for Radiation Oncology & Biology, Department of Oncology, University of Oxford, UK; d Oxford Centre for Functional MRI of the Brain, University of Oxford, UK

**Keywords:** Amide proton transfer, Chemical exchange saturation transfer, Magnetization transfer, Bloch–McConnell equations

## Abstract

Many potential clinical applications of chemical exchange saturation transfer (CEST) have been studied in recent years. However, due to various limitations such as specific absorption rate guidelines and scanner hardware constraints, most of the proposed applications have yet to be translated into routine diagnostic tools. Currently, pulsed CEST which uses multiple short pulses to perform the saturation is the only viable irradiation scheme for clinical translation. However, performing quantitative model-based analysis on pulsed CEST is time consuming because it is necessary to account for the time dependent amplitude of the saturation pulses. As a result, pulsed CEST is generally treated as continuous CEST by finding its equivalent average field or power. Nevertheless, theoretical analysis and simulations reveal that the resulting magnetization is different when the different irradiation schemes are applied. In this study, the quantification of important model parameters such as the amine proton exchange rate from a pulsed CEST experiment using quantitative model-based analyses were examined. Two model-based approaches were considered – discretized and continuous approximation to the time dependent RF irradiation pulses. The results showed that the discretized method was able to fit the experimental data substantially better than its continuous counterpart, but the smaller fitted error of the former did not translate to significantly better fit for the important model parameters. For quantification of the endogenous CEST effect, such as in amide proton transfer imaging, a model-based approach using the average power equivalent saturation can thus be used in place of the discretized approximation.

## Graphical abstract

**Graphical abstract:**

**Figure:**
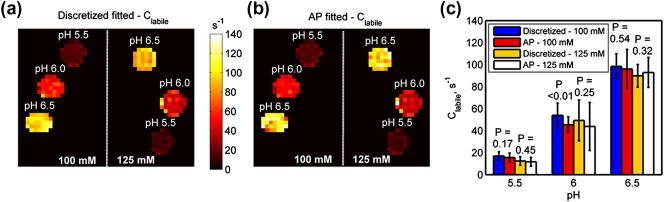

**Highlights:**

► Quantitative model-based analyses (QMAs) were performed on a pulsed CEST experiment. ► Important fitted model parameters (*P*) using different methods were compared. ► Discretization method (slow) could fit the experimental data with smaller error. ► But insignificant difference was found for *P* using continuous approximation (fast). ► Continuous approximation can be used for QMA in amide proton transfer imaging.

## 1 Introduction

Chemical exchange saturation transfer (CEST) is an MRI technique in which saturation is applied at the frequency of exchangeable labile protons with readout being performed from water protons. Through chemical exchange of saturated protons from the labile group to the unsaturated protons in the bulk water, a detectable signal reduction can be measured [1–3]. This mechanism provides an indirect way to detect dilute labile protons that would otherwise be undetectable due to their low concentration. A number of labile proton groups have been investigated for potential clinical translation such as endogenous mobile proteins and peptides in tumor [4–7] and stroke [8–12] diagnosis (amide proton transfer (APT)), hydroxyl groups for type 2 diabetes [13] (glycoCEST), myo-inositol (MI) for Alzheimer’s disease [14] (MICEST), iopamidol – an X-ray contrast agent, for pH mapping of kidney (ratiometric CEST) [15] and exogenous paramagnetic CEST (PARACEST) agents for monitoring brain neuronal activity [16], detecting enzyme activity [17] and as a potential reporter for gene therapy [18].

Currently, there are two irradiation schemes that can be used to perform the saturation: continuous CEST (CW-CEST) and pulsed-CEST. CW-CEST uses a long rectangular radiofrequency (RF) pulse to saturate the protons whereas pulsed-CEST replaces the continuous RF pulse with multiple high intensity but short duration pulses.

The CEST ratio (CESTR) [19] or also referred to as magnetization transfer ratio asymmetry (MTR*_asymmetry_*) is the most commonly used metric to measure the CEST effect. It is a form of asymmetry analysis defined as [*I*(−*ω*) − *I*(*ω*)]/*I_o_*, where *I*(*ω*) and *I*(−*ω*) are the measured intensity at the resonance frequency of the labile protons and its mirror frequency about the water resonance, respectively, and *I_o_* refers to the intensity of the reference image in the absence of saturation. However, CESTR depends on experimental parameters such as RF power [20] and saturation time [21]. Moreover, the calculated *in vivo* CESTR includes not only the CEST effect, but also direct saturation of water protons, fat/lipid saturation which causes artifact such as banding around [22] or through [23] the brain, magnetization transfer (MT) [24] and nuclear overhauser enhancement (NOE) effects [2,25]. These factors complicate the quantitative analysis of the CEST effect using CESTR, highlighting the need for a model-based approach to separate these effects.

Unlike the CESTR calculation which only relies on two saturation frequencies, the model-based approach fits a model of the CEST process to the data collected from a range of saturation frequencies (*z*-spectrum). The model is based on the Bloch equations modified for exchange, often referred to as the Bloch–McConnell equations [26,27]. The simplest model-based analysis of CEST effect consists of two pools: water and amide protons; more pools can be added to the analysis to model the various extra effects observed *in vivo*. By having a separate pool for each confounding factor in the CEST experiment, a pure CEST effect can be determined from the data correcting for the confounds.

A shift of water center frequency away from the expected value is a common problem in an MRI experiment, particularly in CEST imaging where this shift will mean that any applied saturation is not necessarily occurring at the offset relative to water that is specified. This is caused by inhomogeneity of the main field and correcting the shift is mandatory to avoid non-negligible errors in a quantitative CEST study. Water saturation shift referencing (WASSR) [28] is one of the most commonly used techniques to correct for this shift; however, the method requires extra scans possibly before and after the CEST imaging. Using a model-based approach eliminates the additional scan(s) required because the shift can be determined directly from the collected spectrum as part of the model fitting [29].

Performing model-based quantitative analysis of the CEST effect for CW-CEST is simple and is generally achieved using the analytical solution to the Bloch–McConnell equations. However, CW-CEST is not feasible in clinical applications due to specific absorption rate (SAR) and hardware limitations, making pulsed-CEST the only viable irradiation scheme for clinical translation currently. Finding the proton MR behavior in response to time varying RF power as present in the pulsed-CEST scheme for model-based analysis is time consuming because the solution to the Bloch–McConnell equations must be arrived at either using a numerical differential equation solver or discretizing the pulses into a series of short continuous RF segments. In the latter case, referred to here as the discretization method, the individual segments are solved using the simple analytical solution for CW-CEST with the magnetization being propagated through each of the segments, the final values from one segment serving as the initial conditions for the next one [25,30]. Due to the combination of the repeated calculations required in the discretization method and the multiple iterations within the optimization used for model-based strategy, the analysis of pulsed-CEST is often much slower than its continuous counterpart. Hence, pulsed-CEST is often treated as CW-CEST by finding the equivalent average field (AF) [31,32] or power (AP) [33] of the pulse train to perform the analysis using the faster solution to the Bloch–McConnell equations under continuous saturation.

Recently, studies have shown that a continuous approximation (both AF and AP) produces narrower off-resonance excitations when compared with pulsed saturation [33] and that the CESTR is different for pulsed-CEST and CW-CEST when the exchange rate is more than 50 s^−1^ [30]. These raise the issue whether pulsed-CEST can be analyzed via the equivalent CW-CEST or a discretization method must be used.

In this study, the differences in the *z*-spectra from a pulsed-CEST experiment and the equivalent continuous (AF and AP) approximation are examined using simulations to determine the validity of the latter for the analysis of pulsed-CEST data. Additionally, model-based quantitative analysis of pulsed-CEST data from a tissue-like phantom using the continuous approximation and discretization methods are compared. The quantified parameters such as water center frequency shift and amine proton exchange rate are evaluated to determine the extent of the errors introduced by using the continuous approximation when analyzing the pulsed-CEST data.

## 2 Materials and methods

### 2.1 Numerical simulation

#### 2.1.1 Comparison of z-spectra

All the simulated results were generated and processed using MATLAB (Mathworks, Natick, MA, USA). The Bloch–McConnell equations for a two-pool model (water and amine protons labeled as pool w and labile, respectively) were used to stimulate *z*-spectra, assuming a field strength of 4.7 T. A pulsed saturation scheme of 50 Gaussian pulses with flip angle (FA) of 180° and 50% duty cycle (DC) was considered, where each pulse had total duration 40 ms, *T_pd_* (Gaussian pulse + inter-pulse delay). The saturation was performed from −3.8 to 3.8 ppm (−760 to 760 Hz at 4.7 T) with 0.19 ppm (38 Hz) increments. To model pulsed saturation, the discretization method was used with each Gaussian pulse discretized into 1024 segments. Crusher gradients with alternating signs, assumed to have been applied during the inter-pulse delays, were modeled by setting the transverse magnetization to zero at the end of the inter-pulse period. The readout was performed after all the Gaussian pulses had been applied.

The equivalent AF and AP of the Gaussian pulses were calculated using the following formulas [33]: $\text{AF}=1/t*\int_{0}^{t}B_{1}\mathrm{dt}$ and $\text{AP}=\sqrt{\left(1/t*\int_{0}^{t}B_{1}^{2}\mathrm{dt}\right)}$, where *t* is equivalent to the *T_pd_* defined above and *B*_1_ is the RF power amplitude. The continuous z-spectrum was simulated using the continuous saturation solution for 2 s, equivalent to the total saturation time of pulsed-CEST (50 pulses × 0.04 s/pulse).

The remaining variables in the model were set according to published values: longitudinal relaxation times, *T*_1_*_w_* = 3 s, *T*_1_*_labile_* = 1 s; transverse relaxation times, *T*_2_*_w_* = 60 ms, *T*_2_*_labile_* = 8.5 ms [34]; amine proton exchange rate, *C_labile_* = 50 s^−1^; amine proton concentration, *M_labile_*_0_ = 0.33 M and water proton concentration, *M_w_*_0_ = 100 M (equivalent to 0.0033 for the proton concentration ratio, *M_labile_*_0_/*M_w_*_0_).

#### 2.1.2 Minimum discretization required by the discretization method

The computational time required to compute a *z*-spectrum using the discretization method is correlated with the number of segments used to generate a discrete approximation to the pulse shape. In order to aid the comparison of the discretized and continuous approximation for model fitting, the minimum number of segments, *N*, required for the former was investigated to minimize the processing time.

The pulsed CEST effect depends on the pulsed parameters used (FA, *T_pd_*, DC and pulse shape). A range of parameter values was simulated: FA varied from 60° to 300° with intervals of 60°, *T_pd_* = 20, 40, 80, 100 and 200 ms, and DC changed from 0.3 to 0.8 with 0.1 increments. The rest of the parameters used were the same as above.

The Gaussian pulse was discretized into 2*^n^* segments (*n* = 1 to 10) and the 1024 segment result was used as the benchmark. Root mean square (RMS) error between the spectra generated using the reduced number of segments and the benchmark was calculated; the smallest number of segments which had a normalized RMS error smaller than 0.1%, was chosen as *N* for that set of pulsed parameters.

### 2.2 In vitro

#### 2.2.1 Phantom preparation

Tissue-like phantoms were prepared according to Sun et al. [35] using creatine and agarose (Sigma Aldrich, St. Louis, MO, USA). Creatine was added to deionized water first to reach concentrations of 100 and 125 mM. Once the creatine had fully dissolved, agarose was added to form 3% of the mixed solution and then heated to boiling. After that, the mixed solution was maintained at 50 °C and titrated to pH values of 5.5, 6 and 6.5 before being transferred to different 2 ml vials. A plastic container was used to house all the vials and filled up with agar to minimize field inhomogeneity. The phantoms were left to solidify at room temperature prior to the MRI experiment.

#### 2.2.2 MRI experiments and data processing

All the images were acquired using a 4.7 T Varian DirectDrive™ spectrometer (Agilent Technologies, Santa Clara, CA, USA). The main magnetic field (*B*_0_) was shimmed to minimize field inhomogeneity artifacts and the RF field was calibrated before experiments. The pulsed parameters used were identical to the simulation: 50 Gaussian pulses, FA = 180°, *T_pd_* = 40 ms, DC = 50% and saturation frequencies from −3.8 to 3.8 ppm (0.19 ppm increments). Crusher gradients with alternating signs were applied after each irradiation pulse to spoil the residual transverse magnetization. A single-slice spin-echo (SE) echo planar imaging (EPI) readout was used at the end of the saturation, with a field of view (FOV) of 80 mm × 80 mm, matrix size of 64 × 64, slice thickness of 1 mm, bandwidth of 250 kHz, echo time (TE) of 20 ms and repetition time (TR) of 4 s. An unsaturated scan with the same image properties was also acquired as a reference. The CEST data were acquired in about 6 min.

Besides CEST imaging, relaxation time and magnetic field maps were obtained to account for the inhomogeneity in the scan. An inversion recovery sequence with eight inversion intervals from 100 to 6000 ms was used to measure the *T*_1_ relaxation time of the water pool. Six separate SE images with TEs from 23 to 100 ms were measured to determine the *T*_2_ relaxation time of the water pool. The *T*_1_ and *T*_2_ maps of the water pool were obtained by least square fitting of the image intensity against the TI and TE, respectively. WASSR was applied to find the main magnetic field inhomogeneity. The acquisition parameters were the same as for the CEST imaging, except that the FA was set to 61°. A *B*_0_ map was generated by first finding the saturation frequency that recorded the lowest magnetization, then seven saturation frequencies below and above the minimum point were interpolated to intervals of 0.0019 ppm (0.38 Hz). The water center frequency shift was determined using the Maximum Symmetry algorithm [28] based on the interpolated data. The saturation frequency at which the magnetization was minimum was used as the initial value for the search.

All the maps and *in vitro* CEST data were processed using nonlinear least-square curve fitting function, *lsqcurvefit* in MATLAB (Mathworks, Natick, MA, USA). A three-pool model, which consisted of water (w), amine (labile) and MT, was used to fit the collected data. A Gaussian lineshape function, which has been found to be more appropriate for tissue-like phantoms prepared using agarose, was used to model the MT effect [36].

Model-based analyses using the continuous approximation and discretization method were performed on the *in vitro* data. For the later, it was discretized using the *N* found in the simulation. There were 16 variables in the modified Bloch equations for a three-pool model: amplitude of the RF pulse (*ω*_1_ = 2*πB*_1_, *B*_1_ is determined by the FA but will vary in practice due to field inhomogeneity), longitudinal (*T*_1_*_s_*) and transverse (*T*_2_*_s_*) relaxations, proton concentrations (*M_s_*_0_), exchange rates (*C_s_*) and resonance frequency of the pools (*ω_s_*), where *s* refers to each of pools w, labile and MT. However, the *z*-spectrum is not sensitive to some of these variables (*T*_1_*_labile_*, *T*_2_*_labile_*, *T*_1_*_MT_*) and some can be determined relatively accurately prior to the CEST experiment (*T*_1_*_w_*, *ω_labile_*, *ω_MT_*) or calculated from the equilibrium condition, for example, *C_w_*. As a result, only nine variables (*T*_2_*_w_*, *T*_2_*_MT_*, *M_w_*_0_, *M_labile_*_0_, *M_MT_*_0_, *C_labile_*, *C_MT_*, *ω_w_* and *B*_1_) were fitted. Field inhomogeneity was assumed to shift the water center frequency within ±0.2 ppm and to affect the distribution of *B*_1_ around ±10% of the applied FA. Since it is difficult to separate the effect of the amine proton exchange rate (*C_labile_*) and concentration (*M_labile_*_0_) [37,38], the latter was only allowed to vary within ±5% of literature values derived from similar phantoms [34,39]. Although *T*_2_*_w_* and *M_w_*_0_ could be determined using the multiple TE acquisition scheme and from the unsaturated data respectively, they were still treated as parameters to be fitted (within ±20% of the measured values). The search ranges of the properties of the MT pool (*T*_2_*_MT_*, *M_MT_*_0_ and *C_MT_*) were set according to Zu et al. [33], who used the same phantoms. The remaining variables were assumed to be constant: *T*_1_*_labile_* = 1 s, *T*_2_*_labile_* = 8.5 ms, *T*_1_*_MT_* = 1 s, resonance frequency of amine protons, *ω_labile_* = 1.9 ppm + *ω_w_* [34], resonance frequency of MT pool, *ω_MT_* = *ω_w_* [27] and *T*_1_*_w_* was determined using the inversion recovery sequence.

The sum of square residual and coefficient of determination, *R*^2^, using discretized and continuous model fitting were calculated to assess the goodness of fit. The fitted *ω_w_* using the model-based methods were compared with the WASSR results to study the discrepancies between them. A two-tailed *t*-test was performed on the quantified *C_labile_* using the different approaches to examine whether the estimated parameter values varied significantly. The coefficient of variation (CV) (standard deviation divided by the mean) of the fitted *C_labile_* was also calculated to assess the performance of the different model fitting approaches.

## 3 Results

### 3.1 Numerical simulation

**Fig. 1:**
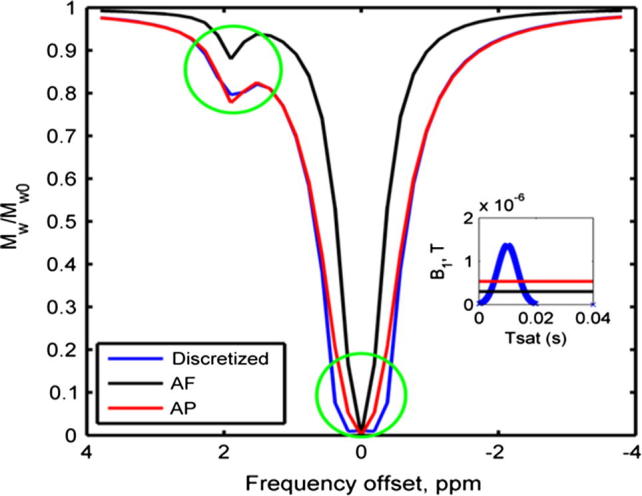
Simulated *z*-spectra using continuous approximation (AF and AP) and discretization method. The light green circles highlight the major differences in the off resonance excitation between different methods. The right inlet plot shows a single Gaussian pulse with its equivalent AF and AP.

The *z*-spectra generated using the discretization method and its continuous approximation (AF and AP) are shown in Fig. 1. The AF approximation was a poor match to the pulsed spectrum; it underestimated the saturated magnetization across the simulated offsets. The *z*-spectrum generated using the AP approximation matched well the spectrum produced by the discretization method, except at the frequency offsets near the water center frequency (0 ppm) and chemical shift of amine protons (1.9 ppm), indicated by the green^1^ circles. Consequently, only the AP continuous approximation was used to perform the continuous model fitting for the phantom data.

**Fig. 2:**
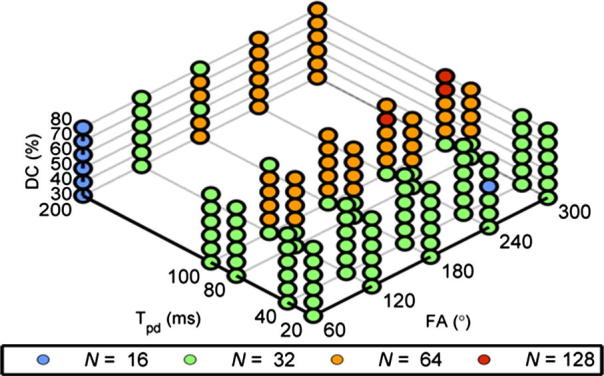
The minimal number of discretization, *N*, required to achieve normalized RMS error to be less than 0.1% when compared with the benchmark magnetization (1024 segments) for different set of pulsed parameters at 4.7 T. This four dimensional relationship (FA (*x*), *T_pd_* (*y*), DC (*z*) and *N* (color)) can be read by following the grid lines along each axis: there will always be 6 ‘balls’ along the *z* direction which represent DC values from 30% to 80%.

Fig. 2 shows the values of *N* required for different pulsed parameters (FA, *T_pd_* and DC) to achieve a normalized RMS error that was less than the threshold (0.1%). The smallest and largest number of segments needed within the investigated pulsed parameter ranges was 16 and 128, respectively. For the set of pulsed parameters used in the *in vitro* study, 32 segments per pulse were found to be sufficient.

### 3.2 In vitro

**Fig. 3:**
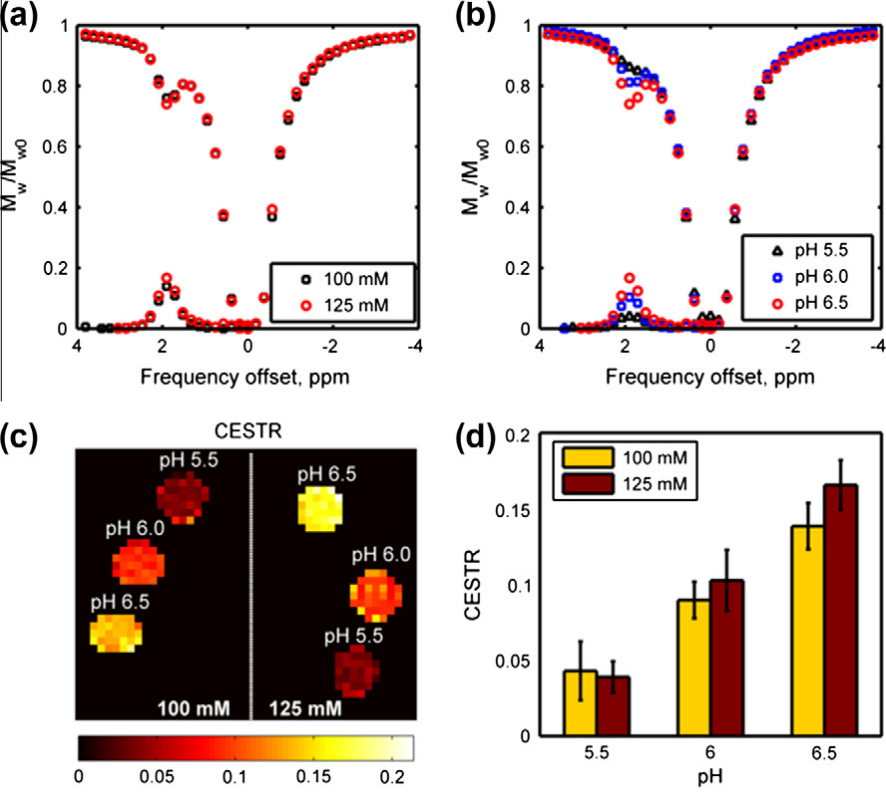
(a) Measured *z*-spectra of different creatine concentration phantoms at pH 6.5. (b) Measured spectra of 125 mM creatine phantoms with different pH values. The asymmetry analysis spectra are plotted underneath the *z*-spectra in each plot. (c) CESTR image and (d) its corresponding error bar plot.

The measured *z*-spectra corrected using the WASSR *B*_0_ map for different creatine concentrations and pH values are shown in Fig. 3a and b, respectively. Fig. 3c shows the CESTR of the phantoms after *B*_0_ correction using the WASSR map and its corresponding error bar plot is presented in Fig. 3d. When either creatine concentration or pH value increased, the dip of the amine pool and CESTR became bigger. The largest CESTR recorded was 16.7% for the 125 mM creatine phantoms with pH 6.5.

**Fig. 4:**
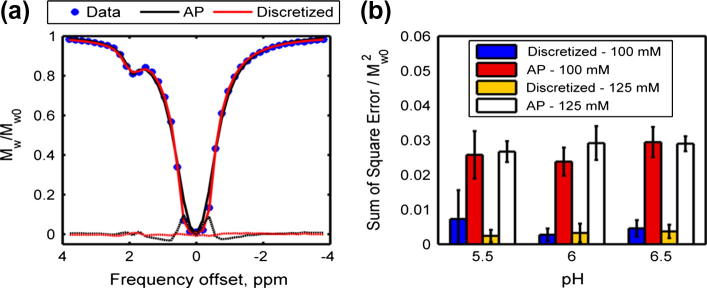
(a) Measured data of phantom with 125 mM creatine concentration at pH 6.0 and continuous (AP) and discretized model fitted spectrum. The residuals are plotted below the fitted spectra (dotted lines). (b) Normalized sum of square error plot of continuous (AP) and discretized model fitting for phantoms with different pH values and concentrations.

*R*^2^ values calculated using *N* sufficient to assure accuracy obtained from the simulation for the discretized model fitting on the phantom data are shown in Table 1. Excellent fits were found for all the measured CEST data (*R*^2^ > 99%). The fitted spectra using continuous and discretized model-based approach for 125 mM creatine phantom at pH 6 are shown in Fig. 4a. The discretization method was able to fit the measured data with small residual errors at all saturation frequencies. Similarly to the simulated data in Fig. 1, the AP continuous method also fitted with small error, except near *ω_w_*. The fitted errors using the discretization method were substantially lower than their continuous (AP) counterparts for all the phantom data, as shown in the normalized sum of square error plot in Fig. 4b.

**Fig. 5:**
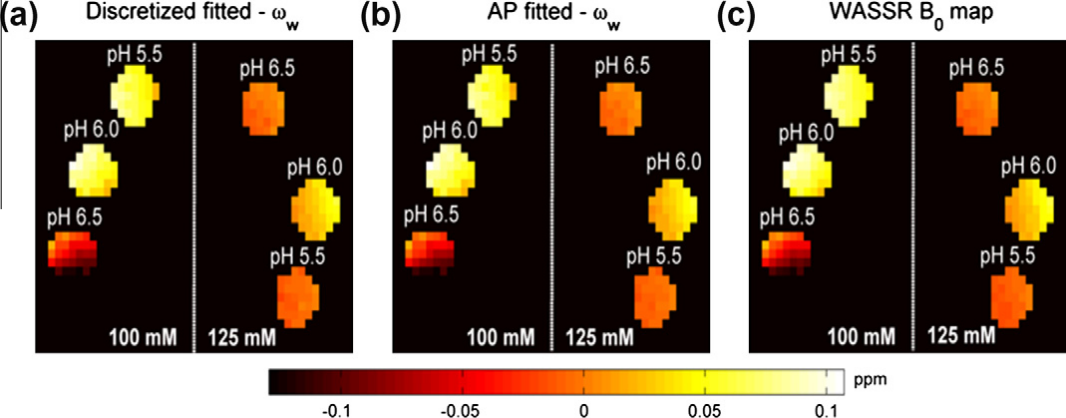
Fitted values of water center frequency, *ω_w_*, using (a) discretized and (b) AP continuous model-based analysis, (c) is the *B*_0_ map generated using WASSR.

Fig. 5 shows the fitted values of water center frequency shift, *ω_w_*, calculated using the discretized and continuous model-based approaches. The results matched well to each other and also to the *B*_0_ map generated using WASSR. The RMS errors and maximum difference found when the model fitted *ω_w_* were compared with the WASSR map were about 1 and 2 Hz, respectively, for both methods.

**Fig. 6:**
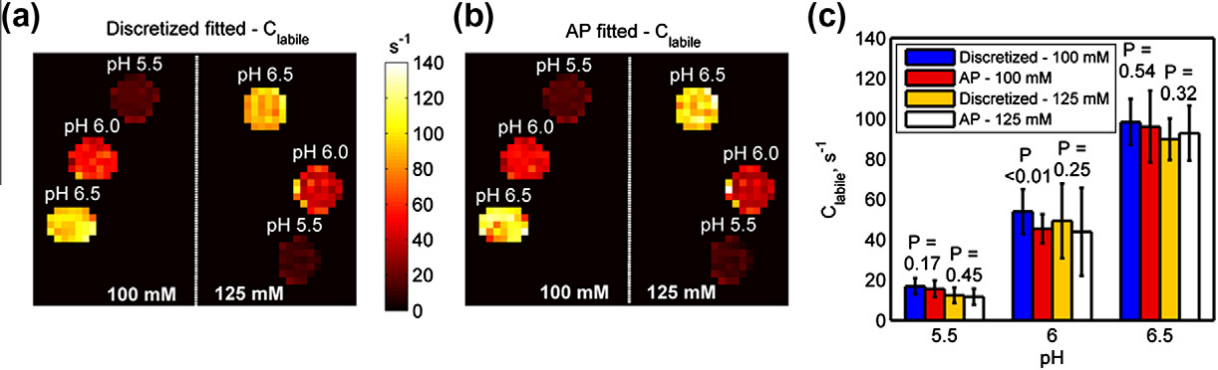
Fitted values of amide proton exchange rate, *C_labile_*, using (a) discretized and (b) AP continuous model-based analysis. The error plot of fitted *C_labile_* using different methods is shown in (c). The *P* values displayed above the bars are the results of the two-tail *t*-tests (*P* < 0.05).

Quantification of amine proton exchange rates, *C_labile_*, using the continuous and discretized model-based approaches is shown in Fig. 6. The difference in the CV of the fitted results (CV*_AP_* – CV*_discretized_*) are shown in Table 2, where positive values indicate the discretized fitted results had smaller variation than the continuous ones. All the CV differences were positive except the results for the phantom with 100 mM creatine concentration at pH 6; these indicated that the discretized model fitting was able to quantify the parameter with higher precision. However, when two-tail *t*-tests were performed on the fitted results, no significant difference was found at 5% significant level, except for the 100 mM creatine concentration at pH 6 (Fig. 6c).

## 4 Discussion

This study illustrates the differences in *z*-spectra obtained using continuous and pulsed saturation, and how these discrepancies can affect the quantified parameters such as *ω_w_* and *C_labile_* using continuous and discretized model-based analysis. As suggested by Zu et al. [33], the differences are caused by the irradiation schemes used, where CW-CEST is able to saturate the protons more efficiently, leading to narrower off-resonance excitation around the frequency offset of water and amine protons, as shown in the simulated (Fig. 1) and measured (Fig. 4a) results.

It is apparent from Fig. 4b that the discretized model-based approach was able to fit better than its continuous (AP) counterpart but the smaller fitted errors of the former did not translate to significantly better quantification of *ω_w_* and *C_labile_*, as shown in Figs. 5 and 6. Although AF is not suitable for fitting the *z*-spectrum, one of the reviewers suggests that the magnitude of its CESTR may be approximately equal to the CESTR calculated from AP for certain pulsed parameters and labile proton exchange rate. The quantified results of *ω_w_* verify that model-based analysis can be used to determine water center frequency shift due to field inhomogeneity and that the additional WASSR scan is not necessarily required when the full *z*-spectrum is available.

When the two-tailed *t*-test was performed for the estimated *C_labile_* for 100 mM creatine phantom at pH 6, the quantified parameter (*C_labile_*) using different model-based approaches was found to be significantly different, as shown in Fig. 6c. This may be caused by the strong correlation between each of the following factors: *T*_2_*_w_* [25], FA and *M_labile_*, with *C_labile_*. The influence of *T*_2_*_w_* and FA on *C_labile_* is significant because the resonance frequency of the amine protons investigated is just 1.9 ppm away from the water protons. *M_labile_* was estimated from the literature and derived from the equilibrium condition which makes the absolute quantification of each of them (*C_labile_* and *M_labile_*) difficult. As suggested by Sun et al. [38], performing model fitting on data measured from multiple RF saturation magnitudes may be one of the ways to achieve independent quantification of the previous two parameters because optimal RF power varies strongly with *C_labile_* but has minimal dependence on *M_labile_*. However, this is not the scope of this study as only a single *B*_1_ was used to perform the saturation. When model fitting was performed on the collected CEST data, all these parameters (*T*_2_*_w_*, FA, *M_labile_*_0_ and *C_labile_*) were allowed to vary, the strong correlation between them might have contributed to the significantly different result in the quantification of *C_labile_*.

Conventionally, the saturation frequencies of CEST are uniformly distributed across the investigated range. However, this kind of sampling schedule will contain samples that are minimally informative to the parameters of interest. For example, *C_labile_* changes mainly affect saturation frequencies near the chemical shift of the exchangeable protons (around 1.9 ppm in this study). Recently, an optimal sampling schedule (OSS) [40] was introduced to maximize the information for the parameters of interest from the measured data. OSS selects the saturation frequencies based on the parameter sensitivity functions which describe how sensitive the data are to changes in the parameter values at a particular saturation frequency. When an OSS was optimized for *ω_w_*, *M_labile_*_0_ and *C_labile_*, the algorithm proposed a schedule that sampled repeatedly around the water center frequency and the chemical shift of the exchangeable protons with minimal or no samples at the other frequency offsets. By doing so, better signal to noise ratio data are achievable, resulting in an improvement in the accuracy of the important parameters estimated from the model fitting. The results of this study, namely those in Figs. 1 and 4a, indicate that the predominant differences between the pulsed and continuous *z*-spectra occur around the two resonances which coincide with the frequency offsets most sampled by the OSS. This might imply that quantitative analysis of data acquired using pulsed-CEST with an OSS strategy may not be feasible with the continuous approximation and in this case the discretization method has to be used.

In practical data analysis scenarios, the results in Fig. 2 indicate that the number of discretized segments required by the discretization method varies according to the pulsed parameters used and could be reduced from the benchmark (1024 segments) to minimize the computational cost. Previously, analysis has been performed by discretizing each pulse into 64 [30] or 512 [25] segments. The computation time required to calculate a spectrum using 512 segments per pulse was roughly 16 times (9.8 min/0.629 min) longer than 32 segments per pulse used in this study and 4 (9.8 min/2.483 min) times longer than the largest discretization needed for the range of pulsed parameters simulated. The computational time reduction above was recorded from an Intel Xeon CPU E5520 @ 2.27 GHz with 8G of RAM. When discretized model fitting, which requires iterative calculation of the magnetization, is applied, using a smaller number of discretized segments is especially important as it will result in a substantial reduction in computational cost. Despite the reductions in computational costs afforded by the reductions in the number of discretization required in practice, analysis of pulsed-CEST data using a discretized pulse train is still high compared to the continuous equivalent (a few seconds to calculate a spectrum per iteration).

APT imaging relies on the exchange of protons between the endogenous amide mobile proteins in the composite backbone with the bulk water to produce contrast at the cellular protein level. The APT/CEST effect observed *in vivo* is small due to the low concentration of the proteins and the endogenous amide protons involved in the chemical exchange have slow exchange rates [8]. When an evenly distributed sampling schedule and a pulsed irradiation scheme are used in the APT imaging, the results of phantoms with pH 5.5 in Figs. 5 and 6 suggest that AP continuous model-based approach can be applied in place of the computationally expensive discretization method in the quantitative study, assuming the difference of the resonance frequency of amine and amide protons has negligible effect. Since the endogenous amide protons have slow exchange rates and their resonance frequencies are further away from the water resonance when compared to amine (smaller direct saturation effect), it is highly probable that the difference should have minimal or no effect on the quantitative fitting results.

**Fig. 7:**
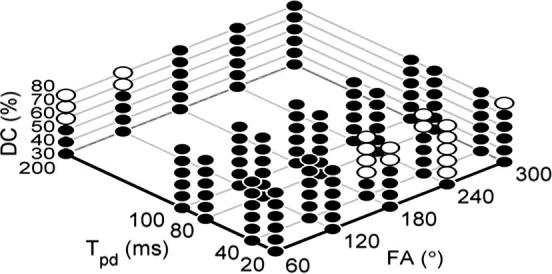
The other sets of pulsed parameters which should produce equivalent quantitative fitting results (white circles) for the important model parameters investigated when AP approximation is used. Black circles represent the sets of pulsed parameters that had sum of square and CESTR difference bigger than the benchmark (the one used in this study).

In order to broaden the applicability of this study to a wider range of acquisition strategies and parameter values, additional simulations were performed by comparing the sum of square and CESTR difference of the simulated *z*-spectra generated by AP and the discretization method, taking the results from the phantom study as the benchmark. Any other set of pulsed parameters which produced sum of square and CESTR difference smaller than the benchmark should also be able to produce the same quantitative fitting results. The pulsed and model parameter values used to generate the results in Fig. 2 were investigated, except *C_labile_* was set to be 28 s^−1^ which was the amide proton exchange rate found in APT imaging. The result is presented in Fig. 7, where white circles refer to the sets of pulsed parameters which had smaller sum of square and CESTR difference than the benchmark and black circles represent the opposite. Since the investigated differences were smaller than the benchmark, these sets of pulsed parameters should also be able to generate equivalent quantitative fitting results for the important model parameters when the continuous approximation is used.

However, using AP continuous approximation to replace discretization method may not be translated to a pulsed CEST experiment that involves high exchange protons such as PARACEST agents because CESTR has been observed to be different between CW-CEST and pulsed-CEST when *C_labile_* is higher than 50 s^−1^ and when the exchange rate increases further, the difference becomes larger [30]. For the pulsed-CEST study in this higher exchange regime, the discretization method may need to be applied for more accurate data fitting and model-based quantitative analysis.

There are multiple effects or metabolites such as amide, MI, NOE, fat and MT that can affect the *in vivo* CEST experiment. In order to separate these confounding effects or metabolites and model the *z*-spectrum accurately for quantitative model-based analysis, each of them should be treated as a separate pool. A three-pool model which consists of water, the labile protons of interest and MT may be the minimum required to model the *in vivo* environment. However, the *z*-spectra acquired at 7 T reveal a broad group of resonances between 0 and 5 ppm, and appreciable saturation effects observed between 0 and −5 ppm [29]. Thus, it is possible that a three-pool exchange model would be insufficient to perform the quantitative model-based analysis on a full *z*-spectrum. Having multiple pools in the model-based analysis is a challenging task even when the AP continuous approximation is used because the computational cost of matrix exponential in the analytical solution increases exponentially with the number of pools. Furthermore, increasing the number of pools in the analysis requires that more parameters have to be fitted from the data, leading to higher risk of over-fitting and thus inaccurate results. The OSS discussed above may be one possible solution, since by selectively saturating certain frequency offsets, the contaminations from other labile pools can be avoided. Other simplified analytical approximations to the model solutions such as the relationship in [19] could also be considered, assuming that the inaccuracies introduced by the simplification can be acceptably accounted for. It is believed that the applicability of this study will still hold if the *in vivo* environment can be modeled accurately for slow exchanging protons.

## 5 Conclusions

Studies on tissue-like phantoms with slow exchanging protons saturated by a series of short Gaussian pulses show no significant difference for the important fitted model parameters such as water center frequency shift and amine proton exchange rate when quantitative model-based analysis using either average power approximation or discretization method is used. This suggests that when APT imaging is performed using a pulsed saturation with certain pulsed parameters, the fast continuous approximation (average power) to the time dependent RF irradiation pulses can replace the computationally expensive discretization approach for quantitative model-based analysis.

## Figures and Tables

**Table 1:** Coefficient of determination, *R*^2^ (mean ± standard deviation, %), for discretized model fitting.

Phantom (mM)	pH 5.5	pH 6.0	pH 6.5
100	99.82 ± 0.2	99.93 ± 0.05	99.87 ± 0.07
125	99.94 ± 0.05	99.92 ± 0.06	99.9 ± 0.06

**Table 2:** Difference of coefficient of variation (CV) for the fitted amine proton exchange rate (*C_labile_*) using discretized and continuous (AP) model-based analysis.

Phantom (mM)	CV*_AP_*–CV*_discretized_*^a^		
pH 5.5	pH 6.0	pH 6.5
100	0.0305	−0.0491	0.0690
125	0.0290	0.1217	0.0321

a Positive values mean discretized fitted results have smaller variation.

## Footnotes

1 For interpretation of color in Figs. 1–6, the reader is referred to the web version of this article.
